# Source memory for advertisements: The role of advertising message credibility

**DOI:** 10.3758/s13421-020-01075-9

**Published:** 2020-07-31

**Authors:** Raoul Bell, Laura Mieth, Axel Buchner

**Affiliations:** Department of Experimental Psychology, Heinrich Heine University Düsseldorf, 40225 Düsseldorf, Germany

**Keywords:** Source monitoring, Source credibility, Schematic knowledge, Media trust, Advertising

## Abstract

Advertising is seen as an untrustworthy source because of the perceived self-interest of the advertisers in presenting product information in a biased or misleading way. Regulations require advertising messages in print and online media to be labeled as advertisements to allow recipients to take source information into account when judging the credibility of the messages. To date, little is known about how these source tags are remembered. Research within the source-monitoring framework suggests that source attributions are not only based on veridical source memory but are often reconstructed through schematic guessing. In two experiments, we examined how the credibility of advertising messages affects these source attribution processes. The source of the messages affected judgments of credibility at the time of encoding, but the source tags were forgotten after a short period of time. Retrospective source attributions in the absence of memory for the source tags were strongly influenced by the a priori credibility of the messages: Statements with a low a priori credibility were more likely to be (mis)attributed to advertising than statements with high a priori credibility. These findings suggest that the mere labeling of untrustworthy sources is of limited use because source information is quickly forgotten and memory-based source attributions are strongly biased by schematic influences.

## Introduction

The internet has greatly lowered barriers for publishing, disseminating, and accessing information. Therefore, we are exposed to large quantities of information every day. Some of this information stems from trustworthy sources such as independent and unbiased news agencies or research institutes, but other information comes from partial and biased sources. Biased sources may provide misleading information to influence the recipients in the service of certain agendas. Advertisers are often seen as biased sources because they provide paid content that is designed to influence consumers in favor of the advertised brands. A potential remedy against biased misinformation is to distinguish trustworthy content from untrustworthy content by tagging information from untrustworthy sources and by making source information salient (Dias et al., 2020; Pennycook & Rand, 2020). In the long term these source tags can only be effective when people remember them (Nadarevic & Erdfelder, 2019). This is an exercise in source memory because people do not only have to remember the message but also its source. It cannot be taken for granted that the sources are remembered because source memory – in comparison with memory for the message itself – is often comparatively poor (Johnson, 1997; Johnson et al., 1993). The forgetting of sources may therefore limit the effectiveness of source tags in the long term. Here we examine how well the tagging of an untrustworthy source is remembered as such using the labeling of advertisements as an example.

The idea that untrustworthy sources must be disclosed has already found its way into legal regulations. When people read a newspaper on the internet or watch a TV show, they are not only provided with editorial content (which is supposed to be independent of external influences) but they are also exposed to advertisements and sponsored content. In many countries there are regulations in place to ensure that it is transparent for recipients to gauge whether a message is from an impartial source or from an advertiser (e.g., Federal Trade Commission, 2015). These regulations exist because advertising is not credible as a source for valid information. Source credibility is formed by two components: competence and trustworthiness (Pornpitakpan, 2004). Competence is determined by expertise, that is, the knowledge and capability to make correct judgments. Trustworthiness is determined by the perceived self-interest of the source. When the source’s perceived self-interest in representing a product or a brand in a biased way is high, credibility is perceived as low. Advertising is an untrustworthy source because advertisers have an obvious self-interest to portray a product in a favorable light. The recipients of an advertising message have the right to take the source of the message into account when judging its credibility. Therefore, advertisements should be clearly distinguishable from editorial content (e.g., in layout, location, and language).

To increase advertising effectiveness, advertisers often try to conceal the source of the advertising message. In print and online media, so-called *native advertisements* use a similar language and design to the surrounding editorial content and the two are therefore easily confused (Campbell & Grimm, 2019). Lawmakers combat this type of deceptive advertising by requiring that the advertising nature of a message is always disclosed. According to the US *Federal Trade Commission* (2015), advertisements “are deceptive when they convey to consumers expressly or by implication that they’re independent, impartial, or from a source other than the sponsoring advertiser.” It is required by law that the advertising nature of a message has to be disclosed in close proximity to the advertisement. This rule is often implemented by displaying the label *Advertisement* above the advertising message (the label *Sponsored Content* is also often used).

However, even when the labeling of the message allows recipients to recognize the advertising character of a message at the time of encoding, it can only have an effect in the long term when it is remembered later on. From the source memory literature, we know that source memory, as compared to memory for the message itself, is prone to forgetting (Johnson, 1997; Johnson et al., 1993). It is thus possible to remember the message but not its source. The forgetting of source information has long been recognized as a possible influence on the effectiveness of advertising. The *sleeper effect* refers to the observation that the difference between the influence of trustworthy and untrustworthy sources on consumer behavior dissipates over time (Hovland & Weiss, 1951). In consequence, the impact of a message that was initially accompanied by a discounting cue – such as an untrustworthy source tag – may increase with the passage of time. A possible explanation of this effect is that the message is still remembered while its source can no longer be retrieved because it has become dissociated from the message. This illustrates that source memory is a key factor for understanding advertising effectiveness. It is therefore surprising that only a few studies focused directly on how the source of an advertising message is remembered (for an exception, see Law & Hawkins, 1997). More studies are needed to understand how source attributions for advertising messages are made.

According to the well-researched source-monitoring framework (Johnson, 1997; Johnson et al., 1993), people use a multitude of different (e.g., perceptual, semantic, and affective) cues for making source attributions. Memory-based source attributions are not only derived from the phenomenal characteristics of memory, but also based on inferential processes that act on these memory representations. Therefore, people do not only rely on memory for the specific encoding episode, but also make plausibility judgments that draw on semantic knowledge structures. For instance, people may choose the source that is most plausible given the content of the message, such as when they decide that they must have read a statement in a left-wing newspaper because it is consistent with the newspaper’s political position (Johnson, 1997). These inferential processes take advantage of correlations between certain types of item characteristics and sources in the real world and will therefore perform well in situations that conform to schematic expectations but will lead to misattributions when the characteristics of an encoding episode defy these expectations. The existence of an episodic memory representation (in the sense of a veridical record of the past) is not necessary for making schema based source attributions (Bell et al., in press). Formal mathematical models of source monitoring (Batchelder & Riefer, 1990; Bayen et al., 1996) distinguish between source memory, a process that reflects a veridical record of the source, and source guessing, a process that reflects a schema based reconstruction of the source. Different hypotheses can be formulated about how these processes should be influenced by advertising messages and their source.

With respect to veridical source memory, two conflicting hypotheses can be derived from the literature. According to the Spinozan model (Gilbert et al., 1990), all information represented by the cognitive system is initially assumed to be true. The evidential status of a true statement does not have to be remembered as any statement is already presupposed to be true unless its falsity is explicitly remembered. When people encounter evidence suggesting that the information is not to be trusted, it needs to be tagged as false. The model thus leads to the hypothesis that untrustworthy sources are more important to remember than trustworthy sources. The model has received support in early studies showing that distraction or time pressure at encoding selectively interfered with the encoding of “false” tags (Gilbert et al., 1990, 1993; Koslow & Beltramini, 2002). However, other findings are inconsistent with the model’s predictions (Hasson et al., 2005; Skurnik et al., 2005). Furthermore, an important limitation of earlier studies is that source memory and guessing were not clearly distinguished. By contrast, Nadarevic and Erdfelder (2013) took guessing into account when examining memory for sources that differed in credibility. Specifically, they asked their participants to read statements that were associated with three sources: “Hans” always told the truth, “Paul” always lied, and “Fritz” told the truth half of the time. The results did not support the hypothesis that untrustworthy sources are better remembered than trustworthy sources. Instead, both the trustworthy source and the untrustworthy source were better remembered than the source that did not predict the statement’s credibility. Given that in some contexts truth was better remembered than falsity (Nadarevic & Erdfelder, 2019), the results are most supportive of a context-dependent model of source tagging according to which people prioritize the sources that are most informative given the encoding context. Beliefs such as “You cannot believe anything you read on the Internet” may render credible sources more informative, and thus more important to remember, than non-credible sources. This model thus predicts that source memory should not always be enhanced for the untrustworthy source. Instead, source memory should be highly sensitive to contextual factors. Specifically, participants should prioritize the processing of sources that are most informative in a given encoding context (Nadarevic & Erdfelder, 2019).

Source information is known to be easily forgotten (e.g., Deffenbacher et al., 2006). This may limit the influence of advertising labels on later source attributions. When the source of a message can no longer be retrieved from memory, people have to rely on guessing. Guessing strongly relies on schematic knowledge about the world (Bayen & Kuhlmann, 2011; Bayen et al., 2000; Kuhlmann et al., 2012; Spaniol & Bayen, 2002). When people encounter information that is typical for a specific source, guessing is often biased towards the source that is schematically expected. Given that advertising is perceived as an untrustworthy source (Calfee & Ringold, 1994), statements that are not credible should be attributed to advertising. Statements that are perceived to be credible should, by comparison, be attributed to the advertising source with a much lower probability.

In the present study, we presented participants with product statements. The statements were preselected to be credible or non-credible. At encoding, labels indicated the source of each statement. Some of the statements were labeled as advertising messages while others came from a trustworthy source (press releases of a renowned independent research institute for product testing). The main purpose of the present study was to examine how well participants succeeded in remembering these labels in a subsequent memory test. To take the multiple components of source monitoring into account, measurement models have been developed that allow clearly distinguishing among processes that contribute to source judgments (Bröder & Meiser, 2007). Specifically, we report results pertaining to item recognition, source memory, and source guessing. In the present application, *item recognition* refers to the recognition of the statement as old or new. In principle, the detection of an item as old or new does not require recollection of contextual information, but can be based on familiarity alone. *Source memory* refers to the conditional probability of being able to correctly identify the source (advertising or brand testing) of the statement provided that the statement has been correctly recognized as old. *Source guessing* refers to a bias to attribute a statement to a particular source independent of its true source. Following Nadarevic and Erdfelder (2013, 2019), we used the well-established multinomial source-monitoring model (Bayen et al., 1996) to disentangle these processes (for a review of the literature, see Erdfelder et al., 2009). Validation studies have shown that using this model, it is possible to obtain parameter estimates that selectively capture the latent cognitive processes they were designed to measure (Bröder & Meiser, 2007). Our central hypothesis pertains to the model’s guessing parameter, which represents the reconstructive component of source monitoring: When source memory (in the sense of a veridical recollection of the context details) can no longer be retrieved, the content of a product statement is used to reconstruct its source through schematic guessing. In consequence, statements with low a priori credibility should be more likely to be attributed to advertising than statements with high a priori credibility.

## Experiment 1

### Method

#### Participants

We aimed at recruiting at least 100 participants and continued data collection until the end of the week in which this goal was reached. One participant had to be excluded because of a severe visual impairment and one participant could not complete the study because the computer mouse was defect. The remaining sample consisted of 122 students (100 of whom were female) who were recruited on campus at Heinrich Heine University Düsseldorf. Their age ranged from 18 to 38 years with a mean age of 23 (*SD* = 4) years. Written informed consent was obtained from all participants. Up to five participants were seated in individual cubicles in a quiet room. They wore headphones with high-insulation hearing protection covers to further shield them from any remaining background sounds. With a final sample size of *N* = 122, α = .05, and 160 items in the memory test, we were able to detect small effects of size *w* = 0.03 with a statistical power of 1 – β = .95 in the multinomial analysis of the guessing parameters.

#### Materials

We created 260 statements about products such as *“The snack mini pretzels of Beaxen are made of purely organic ingredients.”* In a norming study, 15 participants rated these product statements on a scale ranging from −3 (highly non-credible) to +3 (highly credible). Participants were instructed to read each statement carefully and to answer spontaneously, without much deliberate reflection. To maximize the difference between the conditions, the 80 statements with the lowest credibility ratings and the 80 statements with the highest credibility ratings were selected for Experiment 1. To illustrate, an example for a statement with low credibility is *“Only the cornflakes from the brand Auve make breakfast irresistible”* while an example for a credible statement is *“Glucose tablets from Delklate performed very well in a standardized comparative test.”* The product statements comprised 260 brand names that were created using the pseudoword generator *wuggy* (Keuleers & Brysbaert, 2010). Examples are *Admel, Bastol, Calta, Daubort, Fuson, Gafet, Hörter,* and *Ibutu*. The brand names were randomly assigned to the product statements.

#### Procedure

**Fig. 1:**
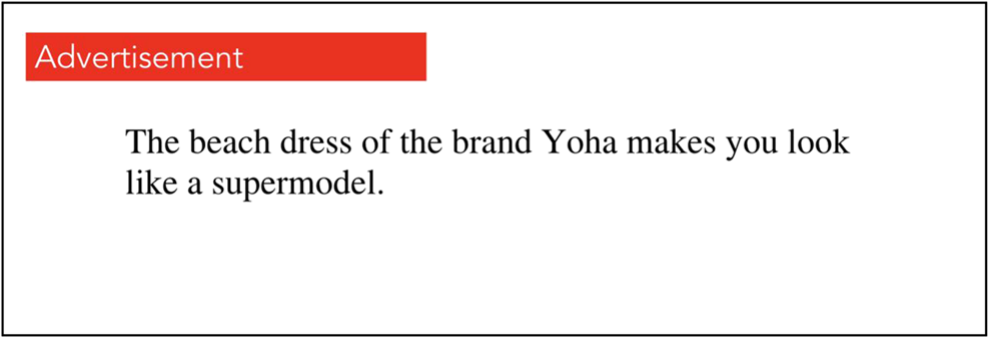
Example for a product statement that was labeled as an advertisement

The encoding of the product statements and their sources was incidental. Participants were instructed that they would see statements about products. Each statement originated from one of two sources: They were either paid advertisements or press releases of a renowned independent (fictitious) institute for product testing (“Foundation for Brand Testing”). In the presentation phase, 80 product statements were shown. Half of these statements had a low a priori credibility and the other half had a high a priori credibility. The statements were written in 32 pt *Times* font. Half of the statements in each condition were assigned to the untrustworthy source and labeled as “Advertisement,” while the other half were assigned to the trustworthy source and labeled “Foundation for Brand Testing.” The labels were shown in white 29 pt *Avenir* font in the upper left corner of the frame in which the product statement was written (Fig. 1). The label “Advertisement” appeared in front of a red background while the label “Foundation for Brand Testing” appeared in front of a blue background to increase the perceptual discriminability of the source tags. For each participant, a different set of 40 product claims with high a priori credibility and 40 product claims with low a priori credibility were randomly drawn from the pool of 160 statements and randomly assigned to the two conditions so that half of the 40 statements of each category were associated with either source. Each participant saw the statement in a different, randomly determined order. The label appeared 1 s before the product statement was shown. The participants were instructed to indicate for each statement how non-credible or credible they thought the statement was given its content and source. The statement’s credibility was rated on a scale ranging from −3 (highly non-credible) to +3 (highly credible). Upon clicking a “continue” button, the statement disappeared from the screen, and the next trial started after an inter-trial-interval of 1 s. A progress bar at the bottom of the screen showed the percentage of trials that had been completed.

Following the presentation phase, 20 trials of serial recall had to be performed as a distractor task. In each trial, eight digits were presented, one after another, for 1 s each. Immediately after the presentation of the digits, eight question marks appeared that had to be replaced by the digits in the order of their presentation, using the number key pad of the computer keyboard. After each trial, participants received feedback about their performance. The distractor task lasted about 10 min.

A surprise source-memory test then followed. Participants saw the 80 statements from the presentation phase, randomly intermixed with 80 new statements, half of which had a low a priori credibility and half of which had a high a priori credibility. All statements were presented in black 30 pt *Arial* font against a white background at the center of the screen. Participants first rated the credibility of the statements on a scale ranging from −3 (highly non-credible) to +3 (highly credible). Then they were asked to indicate whether they had seen the statement before or not by clicking “old” or “new.” After a statement had been classified as “old,” participants were asked to provide a source judgment by indicating whether the statement came from an advertisement or from the Foundation for Brand Testing. This follows the standard procedure of source-memory tests (Bayen et al., 1996). A progress bar at the bottom of the screen indicated the percentage of trials that had been completed.

### Results

#### Credibility ratings

A 2 × 2 × 2 repeated-measures analysis of variance with a priori credibility (high, low), source (advertisement, brand testing), and phase (presentation, test) as independent variables and credibility ratings as dependent variable revealed main effects of a priori credibility, *F*(1,121) = 1358.44, *p* < .01, η_p_^2^ = .92, source, *F*(1,121) = 129.41, *p* < .01, η_p_^2^ = .52, and phase, *F*(1,121) = 125.30, *p* < .01, η_p_^2^ = .51 (Table 1). Statements with high a priori credibility received higher ratings than statements with low a priori credibility, showing that a priori credibility was manipulated successfully. Product statements that were labeled as advertisement received lower credibility ratings than statements that had been labeled as coming from the trustworthy source. On average, the credibility ratings decreased from the presentation phase to the test phase. Phase did not interact with a priori credibility, *F*(1,121) = 1.07, *p* = .30, η_p_^2^ = .01. However, there was an interaction between phase and source, *F*(1,121) = 102.07, *p* < .01, η_p_^2^ = .46. Source had a pronounced effect in the presentation phase but the influence of source was markedly reduced in the test phase. A priori credibility interacted with the source, *F*(1,121) = 12.93, *p* < .01, η_p_^2^ = .10, reflecting the fact that source had a stronger influence on statements with high a priori credibility than on statements that were not credible from the outset. The interaction between phase, a priori credibility, and source was significant, *F*(1,121) = 18.87, *p* < .01, η_p_^2^ = .13, suggesting that the interaction between a priori credibility and source was stronger in the presentation phase than in the test phase. Overall, the data suggest that the influence of the source tags markedly decreased from the presentation phase to the test phase. This may reflect forgetting of the source labels, but note that the credibility ratings likely reflect the joint influence of different types of processes such as item recognition, source memory, and source guessing, so that it is necessary to disentangle these processes via cognitive modeling before drawing conclusions about them.

**Table 1:** Mean credibility ratings as a function of a priori credibility (high, low) and source in Studies 1 (advertisement, brand testing) and 2 (advertisement, unknown, brand testing)

	Experiment 1				Experiment 2			
	High a priori credibility		Low a priori credibility		High a priori credibility		Low a priori credibility	
	*Presentation phase*							
Advertisement	0.66	(0.06)	-1.56	(0.06)	0.71	(0.06)	-1.40	(0.07)
Unknown					0.83	(0.06)	-1.48	(0.07)
Brand testing	1.37	(0.05)	-1.13	(0.07)	1.20	(0.06)	-1.16	(0.08)
	*Test phase*							
Advertisement	0.64	(0.06)	-1.65	(0.06)	0.63	(0.06)	-1.51	(0.07)
Unknown					0.65	(0.06)	-1.54	(0.07)
Brand testing	0.91	(0.05)	-1.46	(0.06)	0.72	(0.06)	-1.49	(0.07)

Values in parentheses represent the standard errors of the means

#### Source attributions

**Fig. 2:**
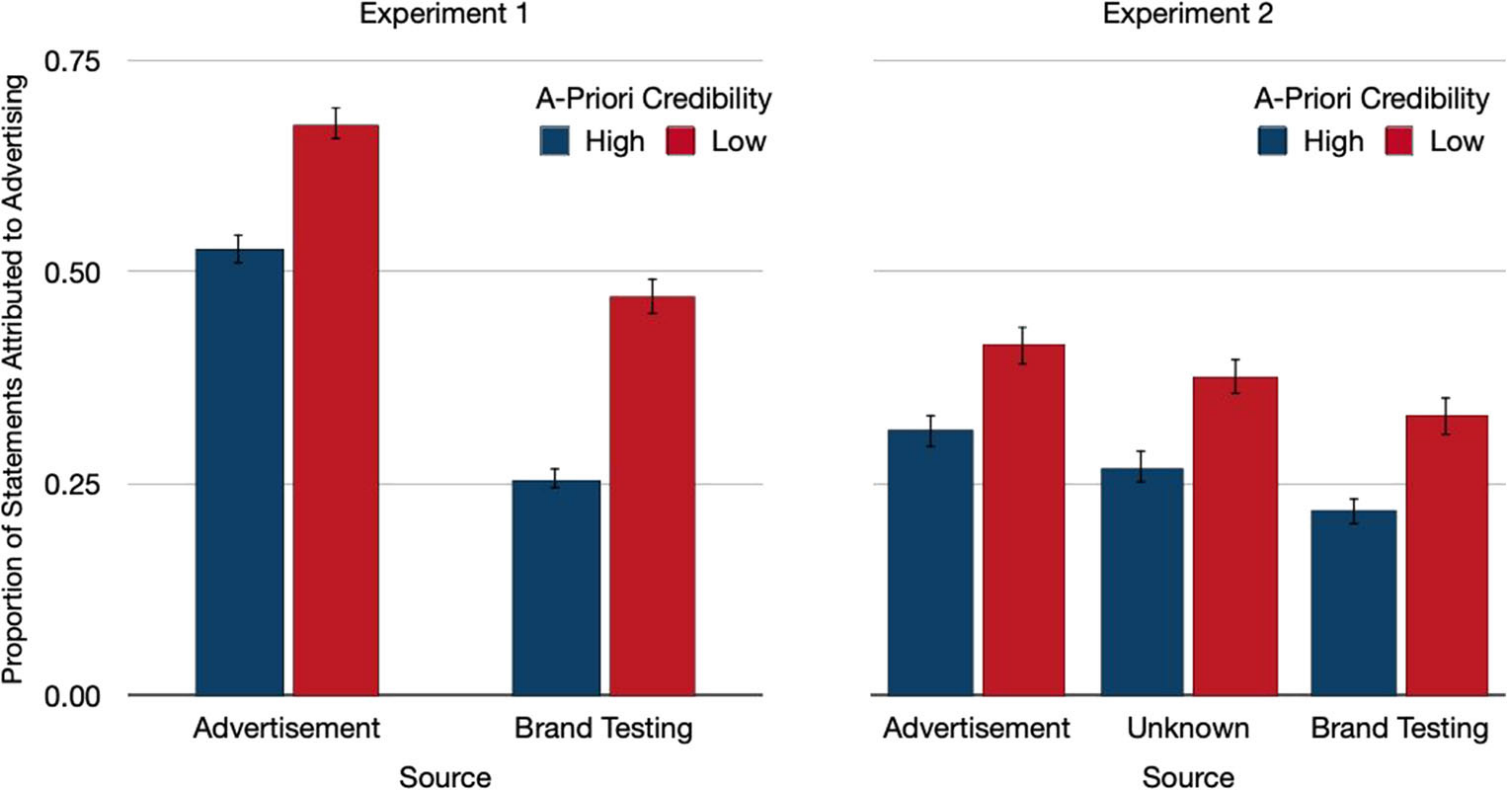
Proportion of statements that were attributed to advertising in the source-monitoring test as a function of a priori credibility and source in Experiments 1 and 2. The error bars represent the standard errors of the means

To analyze the performance in the source-monitoring test, we first assessed the proportion of statements that were attributed to advertising (Fig. 2). Statements that had been labeled as advertisements were more likely to be attributed to the advertising source than statements that came from the trustworthy source, *F*(1,121) = 175.96, *p* < .01, η_p_^2^ = .59, which suggests that participants had some memory for the labels. However, a priori credibility also had a pronounced influence on source attributions, *F*(1,121) = 84.61, *p* < .01, η_p_^2^ = .41, even though there actually was a zero contingency between a priori credibility and source. Statements with low a priori credibility were more likely to be attributed to advertising. This was true both for the statements that were labeled as advertisements, *F*(1,121) = 43.78, *p* < .01, η_p_^2^ = .27, and for the statements of the trustworthy source, *F*(1,121) = 102.53, *p* < .01, η_p_^2^ = .46. The effect was somewhat stronger for the misattributions of the trustworthy statements than for the correct attributions of the advertising statements, *F*(1,121) = 12.96, *p* < .01, η_p_^2^ = .10.

#### Cognitive modeling

**Fig. 3:**
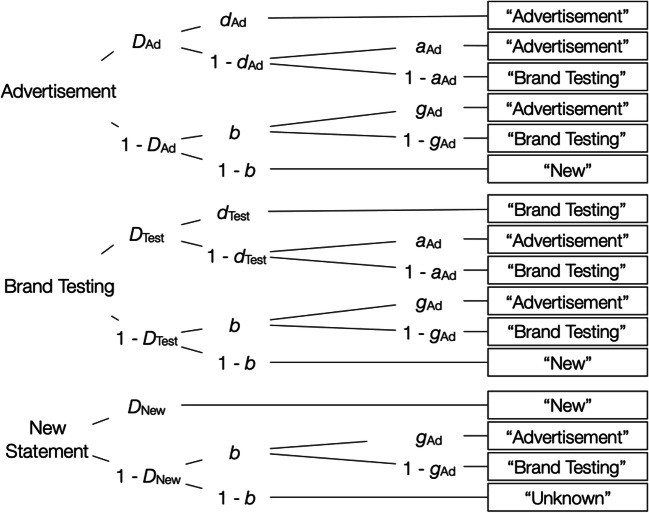
The source-monitoring model for two sources (Bayen et al., 1996), adapted for the present purpose. Each processing tree represents the cognitive processes that occur in response to the items shown on the left side of the figure. The rectangles on the right represent the participants’ answers in the source-monitoring test. Letters along the branches represent the cognitive processes that lead to these answers (*D*• = recognition of an item as old or new, *d*• = source memory, *b* = guessing old, *a*_Ad_ = guessing that a recognized statement had been presented as an advertisement, *g*_Ad_ = guessing that an unrecognized statement had been presented as an advertisement)

Figure 3 shows the source-monitoring model of Bayen et al. (1996), adapted for the present purposes. To illustrate, the first tree of the model depicts the processes that occur in response to product statements that were labeled as advertisements. The parameters represent cognitive processes that may occur with certain probabilities that vary in the [0, 1] interval. With probability *D*_Ad_, these statements are recognized as old. When a statement is recognized as old, participants may also have source memory for the statement with probability *d*_Ad_, in which case the statement is correctly classified as an advertisement. With the complementary probability 1 – *d*_Ad_, participants have no source memory for the statement, in which case they have to guess, with probability *a*_Ad_, that the statement is an advertisement, or, with the complementary probability 1 – *a*_Ad_, that the statement is from brand testing. When the statement is not recognized as old that occurs with probability 1 – *D*_Ad_, participants can still guess that it is old with probability *b*, in which case they may guess that the statement is an advertisement with probability *g*_Ad_ or from brand testing with probability 1 – *g*_Ad_. With probability 1 – *b*, the statement is guessed to be new. Similar processes are thought to occur in response to items labeled “Foundation for Brand Testing” (second tree of Fig. 3), and to new items (bottom tree of Fig. 3). For analyzing the results, we needed two sets of the model trees described above, one for statements with high a priori credibility and one for statements with low a priori credibility. This model is widely used in source-monitoring research to distinguish between source memory and guessing (Bayen & Kuhlmann, 2011; Bayen et al., 2000; Kuhlmann et al., 2012; Schaper et al., 2019). It is well validated as it has been empirically shown that the model parameters allow for an uncontaminated measurement of item recognition, source memory, and guessing (Bayen et al., 1996; Bröder & Meiser, 2007).

The model displayed in Fig. 3 contains eight parameters (*D*_Ad_, *D*_Test_, *D*_New_, *d*_Ad_, *d*_Test_, *b*, *a*_Ad_, *g*_Ad_), each representing the probability with which certain cognitive processes occur. However, there are only six independent data categories to fit, which means that the model is not identifiable. Therefore, equality restrictions have to be imposed on the parameters to obtain an identifiable base model (Bayen et al., 1996). Assumptions that are imposed on the unrestricted model to make it identifiable include (1) the assumption that the probability of detecting an old item as old is identical to the detection of a new item as new, which is the standard assumption of two-high threshold models of item recognition (Snodgrass & Corwin, 1988). Validation studies have found that models incorporating this restriction perform better than alternative models assuming that new items cannot be detected as new and as good as signal-detection based models (Bayen et al., 1996; Schütz & Bröder, 2011; Snodgrass & Corwin, 1988). We therefore included the assumptions that the detection of an item as old did not differ as a function of the source and was equal to the detection of new items (*D*_Ad_ = *D*_Test_ = *D*_New_). To ensure model identifiability, we also included (2) the assumption that source guessing does not differ as a function of the old-new recognition status of the items (*a*_Ad_ = *g*_Ad_), which is a common assumption in studies examining schematic guessing biases (e.g., Bayen & Kuhlmann, 2011; Bayen et al., 2000; Schaper et al., 2019). The base model incorporating these restrictions fit the data well, *G*^2^(2) = 2.95, *p* = .23, which indicates that the assumptions incorporated in the model were compatible with the data (for an additional empirical test of these assumptions, see Experiment 2). The model-based analyses were performed using multiTree (Moshagen, 2010).

##### Item recognition

Item recognition was higher for statements with low credibility than for statements with high credibility, Δ*G*^2^(1) = 47.08, *p* < .01 (Table 2). Implausible or unbelievable statements may stick in memory because of a bizarreness effect (Macklin & McDaniel, 2005).

**Table 2:** Parameter estimates for item recognition (*D*), and source memory for statements coming from advertisements (*d*_Ad_), statements of which the sources were unknown (*d*_Unknown_), or statements associated with the Foundation for Brand Testing (*d*_Test_), as a function of a priori credibility in Experiments 1 and 2. The item recognition parameter *D* refers to the probability of recognizing a statement from the presentation phase as old or a never presented statement as new. The source memory parameter *d* refers to the conditional probability of remembering the source of the statement provided that this statement was recognized as old. The guessing parameter *b* refers to the probability of guessing that an unrecognized item was old rather than new

	Experiment 1				Experiment 2			
	High A priori credibility		Low A priori credibility		High A priori credibility		Low A priori credibility	
*D*	.77	(0.01)	.83	(0.01)	.74	(0.01)	.80	(0.01)
*d*_Ad_	.32	(0.05)	.02	(0.12)	.08	(0.03)	.08	(0.03)
*d*_Unknown_					.04	(0.04)	.00	(0.03)
*d*_Test_	.37	(0.05)	.32	(0.03)	.24	(0.02)	.14	(0.02)
*b*	.21	(0.01)	.33	(0.02)	.23	(0.01)	.33	(0.02)

Values in parentheses represent bootstrapped standard errors

##### Source memory

Overall, source memory was rather poor (Table 2). Descriptively, source memory was somewhat better for the trustworthy source than for the untrustworthy source. The difference between the corresponding parameters was not significant for items with high a priori credibility, Δ*G*^2^(1) = 0.24, *p* = .62, but it was significant for statements with low a priori credibility, Δ*G*^2^(1) = 5.26, *p* = .02.

##### Source guessing

**Fig. 4:**
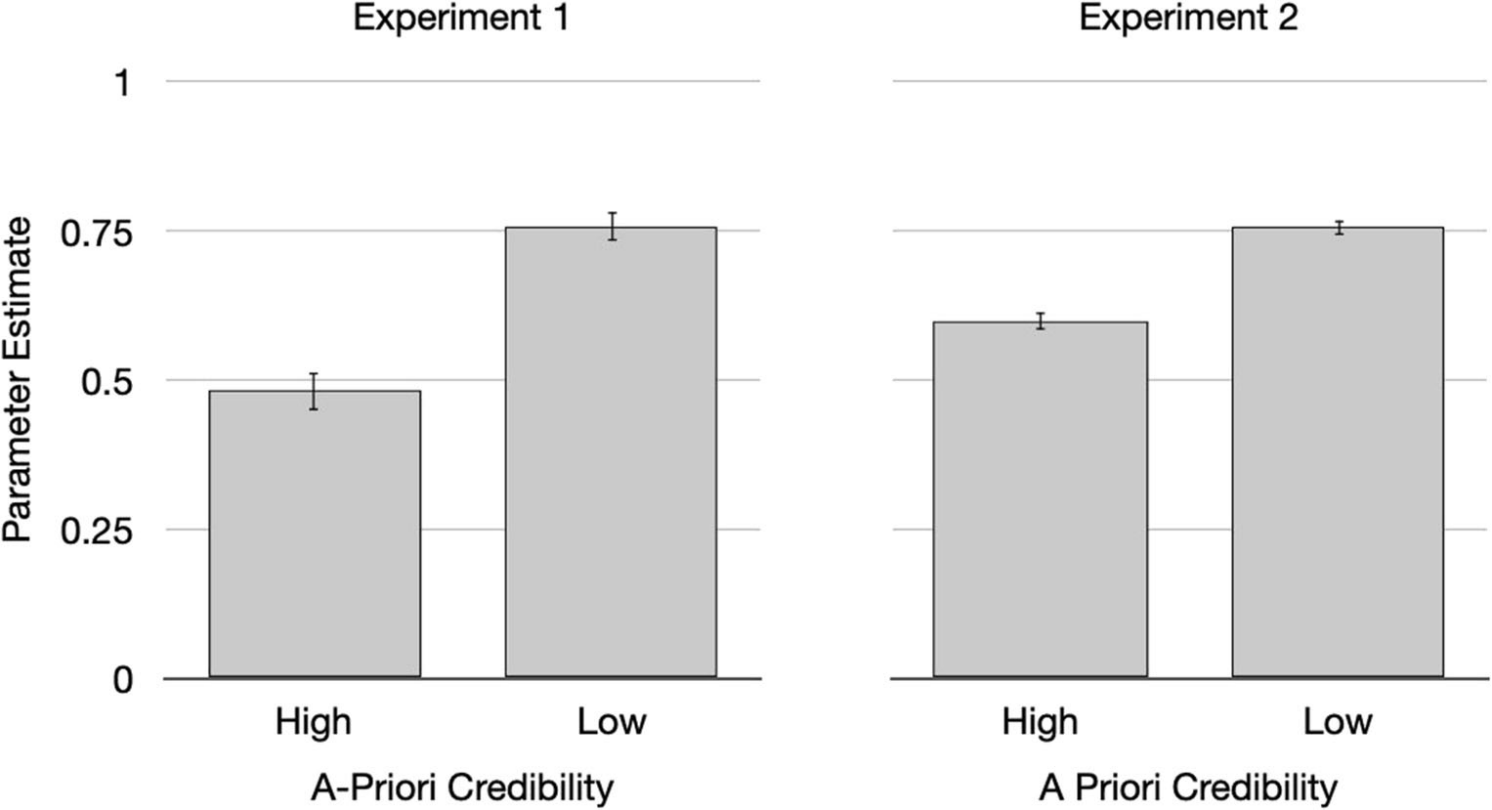
Parameter estimates of the guessing parameter reflecting the probability of guessing that a statement had been presented as an advertisement as a function of the statement’s a priori credibility in Experiments 1 and 2. The error bars reflect bootstrapped standard errors

The source-guessing parameter reflects the probability of guessing that a statement had been labeled as an advertisement (Fig. 4). Statements with low a priori credibility were more likely to be attributed to advertising than statements with high a priori credibility, Δ*G*^2^(1) = 42.14, *p* < .01, confirming that people rely on schematic knowledge to reconstruct the sources when memory fails.

### Discussion

The influence of source tags on credibility judgments decreased markedly from the presentation phase to the test phase. This is most likely due to forgetting of the source tags. In line with this interpretation, source memory was rather poor. The parameter representing the conditional probability of remembering the source tag provided that the statement was still remembered ranged from .02 for the fact that a non-credible statement was an advertising message to .37 for the fact that a credible statement came from a trustworthy source. Participants were thus more likely to forget than to remember the source even after a rather short period of time.

In line with previous studies (Nadarevic & Erdfelder, 2013, 2019), the present results refute the idea that people remember untrustworthy sources particularly well. In the present study, the trustworthy source was remembered even somewhat better than the untrustworthy source. Here this is demonstrated only for particular types of untrustworthy and trustworthy sources (advertisements and brand testing), but the pattern of results fits well with the context-dependent model of source tagging proposed by Nadarevic and Erdfelder (2019). According to this model, trustworthy sources are more informative, and, thus, more important to remember, than untrustworthy sources when people are generally skeptical of new information. Consistent with this idea, the memory advantage for trustworthy sources was numerically stronger – and, in fact, only significant – for statements with low a priori credibility.

When source memory is no longer available due to forgetting, reconstructive guessing processes become increasingly important. Naturally, guessing does not always lead to the reconstruction of the correct source, but may also lead to the misattribution of statements to the false source. As expected, the a priori credibility of the statements had a pronounced effect on these guessing processes. The tendency towards guessing that a statement was an advertising message was much stronger for non-credible statements than for credible statements.

## Experiment 2

The main aim of Experiment 2 was to test whether the results of Experiment 1 can be replicated in an independent study. The design of Experiment 2 was similar to Experiment 1 with one important exception. In Experiment 1, both sources that were used allowed participants to make inferences about the credibility of the product statements because one source (advertisement) was untrustworthy while the other one (Foundation for Brand Testing) was trustworthy. In Experiment 2 we added a control condition in which product statements were presented without any source tag. Statements without source tag provide an interesting control condition against which the other conditions can be compared. This allowed us to test whether the participants are skeptical towards the product statements or believe in the trustworthiness of the statements by default.

### Method

#### Participants

We aimed at recruiting at least 100 participants and continued data collection until the end of the week in which this goal was reached. The final sample consisted of 111 students (71 of whom were female) who were recruited on campus at Heinrich Heine University Düsseldorf. Their age ranged from 18 to 55 years with a mean age of 24 (*SD* = 5) years. Written informed consent was obtained from all participants. Up to nine participants were seated in individual cubicles in a quiet room. They wore headphones with high-insulation hearing protection covers to further shield them from any remaining background sounds. With a final sample size of *N* = 111, α = .05, and 156 items in the memory test, we were able to detect small effects of size *w* = 0.03 with a statistical power of 1 – β = .95 in the multinomial analysis of the guessing parameters.

#### Materials and procedure

Materials and procedure were identical to those of Experiment 1 with the following exceptions. In the presentation phase, 78 product statements were shown. For each participant, 39 statements with high a priori credibility and 39 statements with low a priori credibility were randomly drawn from the same pool of 160 statements used in Experiment 1, and randomly assigned to the three conditions so that 13 of the 39 statements of each category were associated with each of the three sources: One source labeled “Advertisement,” one source labeled “Foundation for Brand Testing,” and one unknown source. In the latter condition, no label was displayed in the upper left corner of the rectangle containing the product statements. In the source-memory test, participants saw the 78 statements from the presentation phase, randomly intermixed with 78 new statements, half of which had a low a priori credibility and half of which had a high a priori credibility. After having classified a statement as “old,” the participants were asked to provide a source judgment by indicating whether the statement came from an advertisement, from the Foundation for Brand Testing, or whether the statement’s source had not been specified.

### Results

#### Credibility ratings

A 2 × 3 × 2 repeated-measures analysis – for which we used the MANOVA approach for repeated measures (O'Brien & Kaiser, 1985) – with a priori credibility (high, low), source (advertisement, brand testing, unknown), and phase (presentation, test) as independent variables and credibility ratings as dependent variable revealed a main effect of a priori credibility, *F*(1,110) = 863.29, *p* < .01, η_p_^2^ = .89. Statements with high a priori credibility received higher ratings than statements with low a priori credibility, showing that a priori credibility was manipulated successfully. There was also a significant main effect of source, *F*(2,109) = 14.49, *p* < .01, η_p_^2^ = .21. Product statements originating from the trustworthy source received higher credibility ratings than statements from the other two sources, *F*(1,110) = 29.19, *p* < .01, η_p_^2^ = .21, but credibility ratings did not differ between statements labeled as advertisement and statements for which the source was not specified, *F*(1,110) = 0.05, *p* = .82, η_p_^2^ < .01. Credibility ratings decreased from the presentation phase to the test phase, *F*(1,110) = 116.77, *p* < .01, η_p_^2^ = .51 (Table 1). Phase did not interact with a priori credibility, *F*(1,110) = 3.86, *p* = .05, η_p_^2^ = .03. However, there was an interaction between phase and source, *F*(2,109) = 23.25, *p* < .01, η_p_^2^ = .30. Source had a strong effect on credibility ratings in the presentation phase but this influence was markedly reduced in the test phase. A priori credibility interacted with source, *F*(2,109) = 4.31, *p* = .02, η_p_^2^ = .07, reflecting the fact that source had a stronger influence on statements with high a priori credibility than on statements with low a priori credibility. There was also an interaction between phase, a priori credibility, and source, *F*(2,109) = 5.26, *p* < .01, η_p_^2^ = .09, suggesting that the interaction between a priori credibility and source was stronger in the presentation phase than in the test phase. The results thus confirm the finding of Experiment 1 that the influence of source on credibility markedly decreased from the presentation phase to the test phase. This may reflect the forgetting of the sources, but conclusions about the underlying memory and guessing processes have to be based on cognitive modeling.

#### Source attributions

As in Experiment 1, we started analyzing performance in the source-memory test by assessing how often statements were attributed to advertising (Fig. 2). There was a main effect of source, *F*(2,109) = 14.33, *p* < .01, η_p_^2^ = .21. Helmert contrasts showed that correct attributions of advertising statements to the advertising source were more likely than misattributions of other statements to the advertising source, *F*(1,110) = 22.88, *p* < .01, η_p_^2^ = .17. Statements that had been presented without a label were more likely to be misclassified as an advertisement than statements that had been presented with a trustworthy label, *F*(1,110) = 13.35, *p* < .01, η_p_^2^ = .11. A priori credibility had a pronounced effect on source attributions, *F*(1,110) = 55.21, *p* < .01, η_p_^2^ = .33, despite the fact that there was a zero contingency between a priori credibility and source. Statements with low a priori credibility were more likely to be attributed to advertising than statements with high a priori credibility, regardless of whether the statements had been labeled as advertisements, *F*(1,110) = 24.68, *p* < .01, η_p_^2^ = .18, had been labeled as coming from the trustworthy source, *F*(1,110) = 33.45, *p* < .01, η_p_^2^ = .23, or had not been labeled at all, *F*(1,110) = 32.90, *p* < .01, η_p_^2^ = .23. There was no interaction between a priori credibility and source, *F*(2,109) = 0.12, *p* = .89, η_p_^2^ < .01.

#### Cognitive modeling

**Fig. 5:**
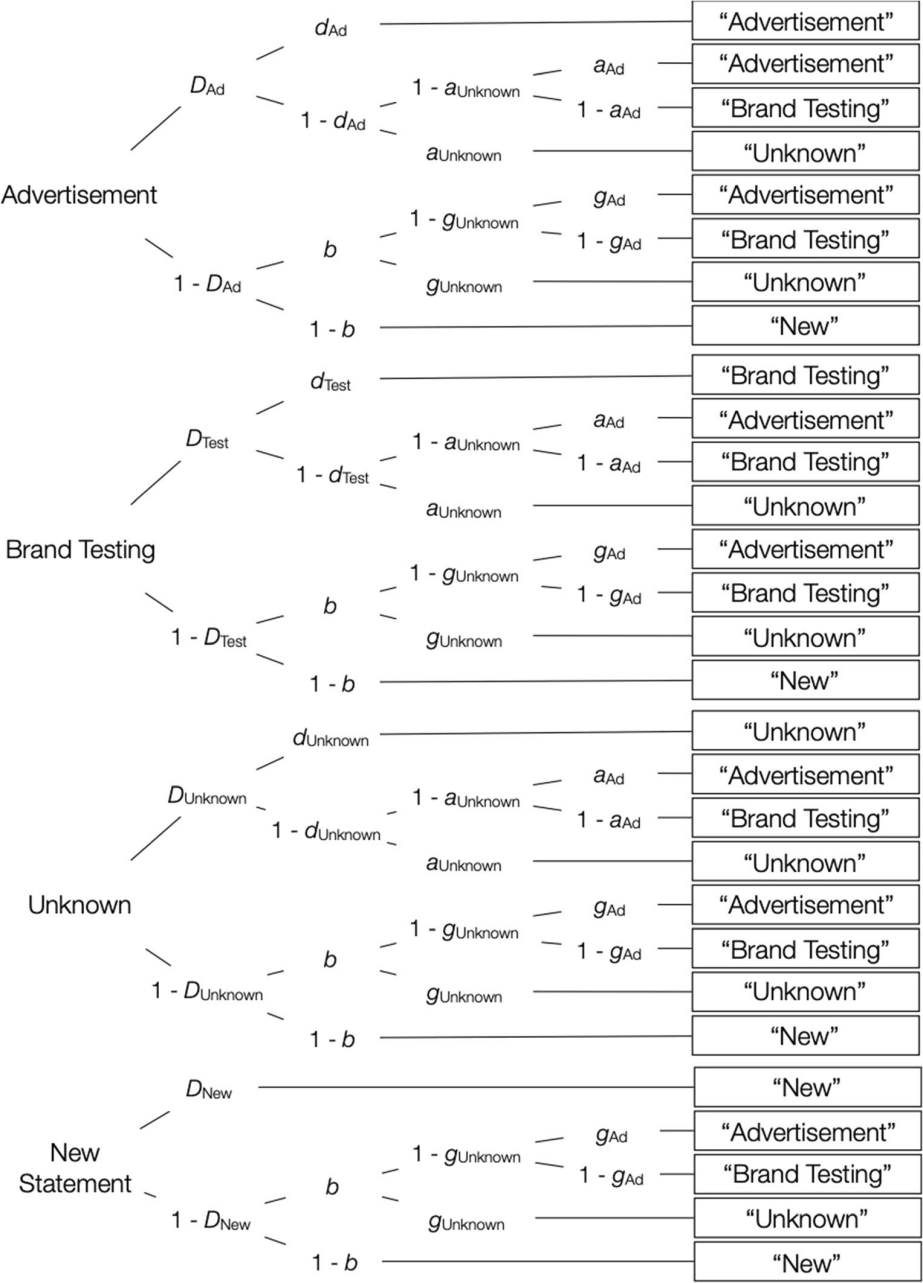
The source-monitoring model for three sources (Keefe et al., 2002), adapted for the present purpose. Each processing tree represents the cognitive processes that occur in response to a specific item. The rectangles on the right represent the participants’ answers in the source-monitoring test. Letters along the branches represent the cognitive processes that lead to these answers (*D*• = recognition of an item as old or new, *d*• = source memory, *b* = guessing old, *a*• = source guessing for recognized old items, *g*• = source guessing for unrecognized items classified as old)

To analyze the results, we used the three-sources variant of the source-monitoring model (Keefe et al., 2002) that is often used in source-monitoring research (Buchner et al., 2009; Kroneisen et al., 2015; Nadarevic & Erdfelder, 2013, 2019). The model, adapted for the present purpose, is shown in Fig. 5. As in Experiment 1, we needed two sets of the model trees, one for statements with high a priori credibility and one for statements with low a priori credibility.

In comparison to the two-sources variant of the model depicted in Fig. 3, the model depicted in Fig. 5 comprises four more parameters (*D*_Unknown_ for recognizing statements whose source was not specified, *d*_Unknown_ for remembering that the source was not specified, *a*_Unknown_ for guessing that the source of a recognized statement was not specified, and *g*_Unknown_ for guessing that the source of an unrecognized statement was not specified). However, there are also six additional independent data categories that generate additional degrees of freedom. We therefore start with a more parsimonious base model that, as a requirement for identifiability, only implies that the detection of items without source information as old is equal to the detection of new items as new (*D*_Unknown_ = *D*_New_). This parsimonious base model fitted the data well, *G*^2^(2) = 2.03, *p* = .36. The assumption that item recognition does not differ as a function of source was compatible with the data, Δ*G*^2^(4) = 2.82, *p* = .59, as was the assumption that source guessing does not differ as a function of whether an item was recognized as old or not, Δ*G*^2^(4) = 3.47, *p* = .48. We then incorporated all of these assumptions into a new base model, which also fitted the data well, *G*^2^(10) = 8.35, *p* = .60. Following the procedure of Experiment 1, this new base model was used as a basis for the hypotheses tests described below. It was also used to obtain the parameter estimates displayed in Fig. 4 and Table 2.

##### Item recognition

As in Experiment 1, item recognition was higher for statements with low a priori credibility than for statements with high a priori credibility, Δ*G*^2^(1) = 35.09, *p* < .01. This may reflect the fact that statements with low a priori credibility made more bizarre claims than statements with high a priori credibility, which may have enhanced memory for these statements (Macklin & McDaniel, 2005).

##### Source memory

Overall, source memory was rather poor (Table 2). Numerically, source memory was somewhat better for the trustworthy source than for the untrustworthy source, but the difference between the corresponding parameters was only significant for statements with high a priori credibility, Δ*G*^2^(1) = 16.20, *p* < .01, but not for statements with low a priori credibility, Δ*G*^2^(1) = 2.73, *p* = .10. While the trustworthy source was better remembered than the unspecified source for both statements with high a priori credibility, Δ*G*^2^(1) = 21.78, *p* < .01, as well as statements with low a priori credibility, Δ*G*^2^(1) = 14.41, *p* < .01, there was only a numerical trend for untrustworthy sources to be better remembered than the unspecified source, but this trend was neither significant for statements with high a priori credibility, Δ*G*^2^(1) = 0.79, *p* = .38, nor for statements with low a priori credibility, Δ*G*^2^(1) = 2.17, *p* = .14.

##### Source guessing

The main focus of the present study lies on the reconstructive component of source monitoring that is reflected in the parameter estimate for the probability of guessing that the statement had been presented as an advertisement (Fig. 4). As expected, the probability of guessing that a statement had been presented as an advertisement was higher for statements with low a priori credibility than for statements with high a priori credibility, Δ*G*^2^(1) = 72.46, *p* < .01, confirming a schematic guessing bias.

### Discussion

As in Experiment 1, there was a decreased influence of source on the credibility judgments from the presentation phase to the test phase. This probably reflects the fact that the labels that were presented together with the statements were quickly forgotten. Consistent with this interpretation, the source-memory parameters reflecting the conditional probabilities of remembering the sources provided that the statements were still remembered ranged from .00 for the fact that a non-credible statement was presented without source tag (indicating that source attributions were *only* due to guessing in this condition) to .24 for the fact that a credible item came from a trustworthy source. These findings confirm that memory for source tags is prone to considerable forgetting even after a short period of time.

The results are inconsistent with the idea that an untrustworthy source is better remembered than a trustworthy source. In fact, source memory for the trustworthy source was slightly better than source memory for the untrustworthy source. Other than in Experiment 1, this trend was numerically stronger – and only significant – for statements with high a priori credibility. The main difference to Experiment 1 is that a control condition was added in which statements were presented without any source information. Given that the statements without source were judged as non-credible (just as the statements from the untrustworthy source), it seems possible that credible statements from the trustworthy source may have stood out from the other statements due to their high credibility and trustworthiness, and were therefore perceived as more informative relative to the other statements. This pattern of findings is consistent with Nadarevic and Erdfelder’s (2019) context-dependent model of source tagging according to which participants do not focus selectively on untrustworthy sources but are instead able to flexibly prioritize information that is most informative in the specific encoding context. When interpreting these results it is important to keep in mind that the present study focuses on advertisements as a specific case of an untrustworthy source. If the aim is to draw general conclusions about memory for trustworthy and untrustworthy sources, conceptual replications with different types of material are desirable. However, broadly consistent findings have also been obtained in paradigms focussing on memory for cheating and trustworthy persons. Here, too, the initial assumption was that memory for untrustworthy sources should be superior to memory for other types of sources (see, e.g., Buchner et al., 2009), but more detailed analyses revealed that human source-memory mechanisms are much more flexile than initially thought (Bell et al., 2012; Kroneisen & Bell, 2013; Kroneisen et al., 2015; Mieth et al., 2016).

When source memory is no longer available due to forgetting, guessing processes become increasingly important. In line with the results of Experiment 1, Experiment 2 confirms that statements with low a priori credibility are more likely to be attributed to the untrustworthy source than statements with high a priori credibility. This finding is in line with schematic guessing (Bayen & Kuhlmann, 2011; Bayen et al., 2000; Kuhlmann et al., 2012; Spaniol & Bayen, 2002).

## General discussion

In many countries regulations exist that require advertisers to disclose the advertising nature of a message in close proximity to it, which gives recipients of the message the opportunity to discount it based on the untrustworthiness of its source (e.g., Federal Trade Commission, 2015). The disclosure of the source is often done by labeling the message as an advertisement or sponsored content. To be effective in the long term, these labels do not only have to be encoded, but they also have to be preserved in memory. The present study examines the perception of, and memory for, product statements that were labeled as advertisements. The results raise doubts about whether the labeling of untrustworthy sources as such is sufficient to allow the recipients of a message to make informed decisions in the long term, at least in situations with high information load, as in today’s digital environments. The influence of the source tags on the credibility judgments decreased markedly from the presentation phase to the test phase. In line with this observation, source information (that is, whether a statement was from a trustworthy or from an untrustworthy source) was forgotten even after a rather short period of time. The high rate of source confusions suggests that the labels have only a transient effect on the participants’ ability to distinguish between information from trustworthy and untrustworthy sources.

It seems noticeable that participants gave low credibility ratings to statements whose sources were not specified or no longer remembered. Such low credibility ratings may indicate skepticism towards information whose sources were unknown. According to the context-dependent tagging model of Nadarevic and Erdfelder (2013, 2019), such a skeptical attitude towards statements with unknown sources should render trustworthy sources more informative, and, thus, more important to remember than untrustworthy sources. In line with these predictions, participants showed a tendency towards remembering the trustworthy source better than the untrustworthy source. Nevertheless, source memory was rather poor overall. Expressed in the conditional probability of remembering the source given that a statement was still remembered, the parameter estimates of source memory did not exceed .37 (Table 2). This was so even though the present experiments provided relatively favorable conditions for source memory in that there was only a short distractor interval between presentation and test and participants devoted their full attention to the stimulus material. In the real world, people are often distracted when skimming through advertisements, and advertising messages have an impact even after longer delays (Shapiro & Krishnan, 2001). In these circumstances, source memory can be expected to be even worse than in the present experiments. One reason why source memory may have been relatively poor is that participants had to process a large amount of information in a relatively short period of time, which is typical for modern-day digital environments in which people are exposed to large quantities of information such as when they browse their Facebook newsfeed or skim through a Google search result list. It remains for future research to test whether people attend more – or maybe even less – to the source of information when they engage with content more deeply such as when they read a full article that is labeled as an advertisement.

When source memory is no longer available, participants fail to distinguish between information from trustworthy and from untrustworthy sources. In consequence, source attributions are primarily determined by reconstructive guessing processes (Bayen et al., 1996). These are strongly affected by schematic knowledge (Bayen & Kuhlmann, 2011; Bayen et al., 2000; Kuhlmann et al., 2012; Spaniol & Bayen, 2002). Due to the low credibility of advertising as a source, statements with low a priori credibility given one’s current knowledge are more likely to be attributed to advertising than statements with high a priori credibility. This aspect of the present results fits well with the findings of Fragale and Heath (2004), who examined how beliefs affect source attributions. In a first study, they changed their participants’ beliefs in urban myths about food contaminations by manipulating the number of times participants were exposed to these myths. When participants had been exposed to a statement multiple times (and thus were presumably more inclined to believe it), the statement was more likely to be attributed to a fact-based print source with high credibility (*Consumer Reports*) than to a tabloid source with low credibility (*National Enquirer*). In a second experiment, participants were shown a newspaper article about a murder. The belief in the guilt of a suspect was manipulated by informing participants that this suspect was linked to the murder by conclusive DNA evidence. Subsequently, statements incriminating this suspect were more likely to be (mis)attributed to a serious newspaper than statements incriminating another suspect. The flip side of this finding is that messages that are not credible – and, therefore, less likely to be believed than messages that are credible – are (mis)attributed to sources with low credibility, as the present study shows. The present multinomial modeling analysis extends previous research by pinpointing the memory and guessing processes that may underlie these belief-based source (mis)attributions.

A practical implication of the present study is that the labeling of advertising statements may have less influence than one might suppose because the labeling is likely to be forgotten even after a comparatively short amount of time. If this finding turns out to be replicable across different applied settings, then advertisers may gain little by trying to circumvent the labeling requirements for advertising. Furthermore, the advertising may be more effective when advertisers pay close attention to the credibility of their advertising claims. Well-tempered advertising messages may not only have the advantage of appearing more credible for the recipients at the time of encoding, but they are also less likely to be attributed to advertising when the source can no longer be retrieved.

While advertisers may want to exploit the limits of source memory, policy makers may want to find ways to prevent this. Native advertising aims at generating confusion between trustworthy and untrustworthy sources. This not only affects the processing of the advertising messages at the time of encoding but also how these messages are remembered. Ultimately, a high rate of confusion between information from untrustworthy and trustworthy sources may negatively affect the belief in information from credible sources (such as news outlets or scientific reports), thereby contributing to a crisis of trust. Therefore, measures must be taken to help consumers to reliably distinguish between trustworthy and untrustworthy sources. The present results suggest that requiring advertisers to label advertising messages as such is only effective in the short term. While such labels allow media users to distinguish between trustworthy and untrustworthy sources at the time of encoding, the source tags are prone to forgetting even after a short period of time, and, therefore, can be expected to have only a limited effect on the impact of untrustworthy information. This may suggest that other ways have to be found to combat deceptive advertising messages that mimic trustworthy sources not only in appearance but also in language and content.

To summarize, the present study focuses on source memory for advertisement, and, specifically, on the factors that determine whether people remember that a product statement is, in fact, an advertisement. The results suggest that the reconstructive nature of source memory has to be taken into account to understand how people distinguish between information from trustworthy and untrustworthy sources in memory.

### Data availability statement

Data and materials of both experiments are available at https://osf.io/kdx63/

## References

[CR1] Batchelder WH, Riefer DM (1990). Multinomial processing models of source monitoring. Psychological Review.

[CR2] Bayen UJ, Kuhlmann BG (2011). Influences of source-item contingency and schematic knowledge on source monitoring: Tests of the probability-matching account. Journal of Memory and Language.

[CR3] Bayen UJ, Murnane K, Erdfelder E (1996). Source discrimination, item detection, and multinomial models of source monitoring. Journal of Experimental Psychology: Learning, Memory, and Cognition.

[CR4] Bayen UJ, Nakamura GV, Dupuis SE, Yang CL (2000). The use of schematic knowledge about sources in source monitoring. Memory & Cognition.

[CR5] Bell R, Buchner A, Kroneisen M, Giang T (2012). On the flexibility of social source memory: A test of the emotional incongruity hypothesis. Journal of Experimental Psychology: Learning, Memory, and Cognition.

[CR6] Bell, R., Mieth, L., & Buchner, A. (in press). Source attributions for detected new items: Persistent evidence for schematic guessing. *Quarterly Journal of Experimental Psychology*10.1177/1747021820911004 10.1177/1747021820911004 32075493

[CR7] Bröder A, Meiser T (2007). Measuring source memory. Zeitschrift fur Psychologie/Journal of Psychology.

[CR8] Buchner A, Bell R, Mehl B, Musch J (2009). No enhanced recognition memory, but better source memory for faces of cheaters. Evolution and Human Behavior.

[CR9] Calfee JE, Ringold DJ (1994). The 70 % majority: Enduring consumer beliefs about advertising. Journal of Public Policy & Marketing.

[CR10] Campbell C, Grimm PE (2019). The challenges native advertising poses: Exploring potential Federal Trade Commission responses and identifying research needs. Journal of Public Policy & Marketing.

[CR11] Deffenbacher KA, Bornstein BH, Penrod SD (2006). Mugshot exposure effects: Retroactive interference, mugshot commitment, source confusion, and unconscious transference. Law and Human Behavior.

[CR12] Dias N, Pennycook G, Rand DG (2020). Emphasizing publishers does not effectively reduce susceptibility to misinformation on social media. The Harvard Kennedy School Misinformation Review.

[CR13] Erdfelder E, Auer T-S, Hilbig BE, Aßfalg A, Moshagen M, Nadarevic L (2009). Multinomial processing tree models. A review of the literature. Zeitschrift fur Psychologie/Journal of Psychology.

[CR14] Federal Trade Commission. (2015). *Native advertising: A guide for businesses*. https://www.ftc.gov/tips-advice/business-center/guidance/native-advertising-guide-businesses

[CR15] Fragale AR, Heath C (2004). Evolving informational credentials: The (mis)attribution of believable facts to credible sources. Personality and Social Psychology Bulletin.

[CR16] Gilbert DT, Krull DS, Malone PS (1990). Unbelieving the unbelievable: Some problems in the rejection of false information. Journal of Personality and Social Psychology.

[CR17] Gilbert DT, Tafarodi RW, Malone PS (1993). You can’t not believe everything you read. Journal of Personality and Social Psychology.

[CR18] Hasson U, Simmons JP, Todorov A (2005). Believe it or not: On the possibility of suspending belief. Psychological Science.

[CR19] Hovland CI, Weiss W (1951). The influence of source credibility on communication effectiveness. Public Opinion Quarterly.

[CR20] Johnson MK (1997). Source monitoring and memory distortion. Philosophical Transactions of the Royal Society of London B.

[CR21] Johnson MK, Hashtroudi S, Lindsay D (1993). Source monitoring. Psychological Bulletin.

[CR22] Keefe RSE, Arnold MC, Bayen UJ, McEvoy JP, Wilson WH (2002). Source-monitoring deficits for self-generated stimuli in schizophrenia: Multinomial modeling of data from three sources. Schizophrenia Research.

[CR23] Keuleers E, Brysbaert M (2010). Wuggy: A multilingual pseudoword generator. Behavior Research Methods.

[CR24] Koslow S, Beltramini RF (2002). Consumer skepticism and the "waiting room of the mind": Are consumers more likely to believe advertising claims if they are merely comprehended?. Advances in Consumer Research.

[CR25] Kroneisen M, Bell R (2013). Sex, cheating, and disgust: enhanced source memory for trait information that violates gender stereotypes. Memory.

[CR26] Kroneisen M, Woehe L, Rausch LS (2015). Expectancy effects in source memory: how moving to a bad neighborhood can change your memory. Psychonomic Bulletin & Review.

[CR27] Kuhlmann BG, Vaterrodt B, Bayen UJ (2012). Schema bias in source monitoring varies with encoding conditions: Support for a probability-matching account. Journal of Experimental Psychology: Learning, Memory, and Cognition.

[CR28] Law, S., & Hawkins, S. A. (1997). Advertising repetition and consumer beliefs: The role of source memory. In W. D. Wells (Ed.), *Advertising and consumer psychology. Measuring advertising effectiveness* (pp. 67-75). Lawrence Erlbaum Associates Publishers.

[CR29] Macklin CB, McDaniel MA (2005). The bizarreness effect: Dissociation between item and source memory. Memory.

[CR30] Mieth L, Bell R, Buchner A (2016). Cognitive load does not affect the behavioral and cognitive foundations of social cooperation. Frontiers in Psychology.

[CR31] Moshagen M (2010). multiTree: A computer program for the analysis of multinomial processing tree models. Behavior Research Methods.

[CR32] Nadarevic L, Erdfelder E (2013). Spinoza's error: Memory for truth and falsity. Memory & Cognition.

[CR33] Nadarevic L, Erdfelder E (2019). More evidence against the Spinozan model: Cognitive load diminishes memory for “true” feedback. Memory & Cognition.

[CR34] O'Brien RG, Kaiser MK (1985). MANOVA method for analyzing repeated measures designs: An extensive primer. Psychological Bulletin.

[CR35] Pennycook G, Rand DG (2020). Who falls for fake news? The roles of bullshit receptivity, overclaiming, familiarity, and analytic thinking. Journal of Personality.

[CR36] Pornpitakpan C (2004). The persuasiveness of source credibility: A critical review of five decades’ evidence. Journal of Applied Social Psychology.

[CR37] Schaper ML, Kuhlmann BG, Bayen UJ (2019). Metamemory Expectancy Illusion and Schema Consistent Guessing in Source Monitoring. Journal of Experimental Psychology: Learning, Memory, and Cognition.

[CR38] Schütz J, Bröder A (2011). Signal detection and threshold models of source memory. Experimental Psychology.

[CR39] Shapiro S, Krishnan HS (2001). Memory-based measures for assessing advertising effects: A comparison of explicit and implicit memory effects. Journal of Advertising.

[CR40] Skurnik I, Yoon C, Park D, Schwarz N (2005). How warnings about false claims become recommendations. Journal of Consumer Research.

[CR41] Snodgrass JG, Corwin J (1988). Pragmatics of measuring recognition memory: Applications to dementia and amnesia. Journal of Experimental Psychology: General.

[CR42] Spaniol J, Bayen UJ (2002). When is schematic knowledge used in source monitoring?. Journal of Experimental Psychology: Learning, Memory, and Cognition.

